# Designing a Randomized Trial with an Age Simulation Suit—Representing People with Health Impairments

**DOI:** 10.3390/healthcare9010027

**Published:** 2020-12-30

**Authors:** Ingo J. Timm, Heike Spaderna, Stephanie C. Rodermund, Christian Lohr, Ricardo Buettner, Jan Ole Berndt

**Affiliations:** 1 German Research Center for Artificial Intelligence (DFKI), Cognitive Social Simulation, Kaiserslautern, Branch Trier, Behringstr. 21, 54296 Trier, Germany; 2 Business Informatics I, Department of Computer Science, Trier University, 54286 Trier, Germany; rodermund@uni-trier.de (S.C.R.); berndt@uni-trier.de (J.O.B.); 3 Department of Nursing Science, Division of Health Psychology, Trier University, 54286 Trier, Germany; spaderna@uni-trier.de; 4 Faculty of Business Studies, Aalen University, 73430 Aalen, Germany; ricardo.buettner@hs-aalen.de

**Keywords:** experimental design, physical activity, fitness tracker, questionnaires

## Abstract

Due to demographic change, there is an increasing demand for professional care services, whereby this demand cannot be met by available caregivers. To enable adequate care by relieving informal and formal care, the independence of people with chronic diseases has to be preserved for as long as possible. Assistance approaches can be used that support promoting physical activity, which is a main predictor of independence. One challenge is to design and test such approaches without affecting the people in focus. In this paper, we propose a design for a randomized trial to enable the use of an age simulation suit to generate reference data of people with health impairments with young and healthy participants. Therefore, we focus on situations of increased physical activity.

## 1. Introduction

It is a well-known problem that the availability of professional caregivers to meet the needs of the current population is restricted [1]. Demographic change will intensify this already challenging situation significantly [2,3,4]. In order to ensure demand-oriented care in the future, the independence of people, for example, with chronic diseases, must be maintained for as long as possible. In Germany, since January 2017 a person’s need for care has been determined on the basis of their independence [5]. Physical activity has an increased influence on the independence of a patient [6] and thus on the quality of life [7,8]. On top of this, physical activity contributes to a decrease in mortality risk [9]. One approach to preserve independence is to promote physical activity using assistance approaches (e.g., by using Internet-of-Things or Ambient Assisted Living technologies). A particular challenge here consists in planning, designing or examining assistance approaches without affecting or even endangering the people to be supported.

The project *SiNuS-Pflege* (*Simulation of Nudging methods to Strengthen the independence of people in need of care*, German: “Simulation von Nudging-Methoden zur Stärkung der Selbstständigkeit Pflegebedürftiger”) investigates, how specific assistance approaches can be tested without involving people with health impairments. A particularly important target group are people with chronic heart failure. However, due to health and ethical considerations participants in this group can only be included in a study to a limited extent. Therefore, a method is needed that represents people with chronic heart failure and the corresponding impairments without actually involving them.

An age simulation suit offers an opportunity to make (age-related) health impairments perceptible to younger people (see, e.g., References [10,11]). Those impairments include physiological (e.g., a reduction of muscle strength of about 25% [12], visual (e.g., an imparoment concerning the near vision) as well as cognitve processes (e.g., a reduction in the ability to do multiple tasks at once [13,14]). Groza et al. identified major components, that an age simulation suit needs to have in order to “simulate natural sensations of ageing” [15]. According to the authors, a *head module* (including manipulation of vision, hearing and neck mobility), a *torso module*, (e.g., to reduce spine movement), a *legs module* (for impairments of hip, knee, ankle and foot movement) and an *arm module* (e.g., reducing tactile sense and shoulder, elbow and wrist movement) are required. In recent years, many age simulation suits have been developed to meet these requirements. For instance, *AGNES* (Age Gain Now Empathy System) developed by the MIT AgeLab is designed to simulate several impairments as visual, motor or flexibility of a person. Therefore, they use an overall besides many different other components as a helmet, glasses or ear plugs [16]. Other age simulation suits make use of an overall with an integrated exoskeleton, that is controlled by a computer to add force on the participants’ joints and thus complicate movement. Additionally, visual and hearing impairments are simulated utilizing a virtual reality helmet including headphones [17].

In the current literature, the use of an age simulation suit mainly focuses on subjective aspects (see Reference [18]), to increase empathy of the users in care professions [10,11] or with the goal of adapting the ergonomics of work places [19]. Focusing on increasing empathy for the elderly, Lee and Teh conduct a randomized controlled study with a homogeneous group of 120 pharmacy students. Here, the intervention group is equipped with an age simulation suit and asked to perform activities of daily life, for example, sitting down on a sofa and stand up again, combing their own hair or picking up a piece of paper [20]. These studies predominately use subjective assessment and self-assessment as scientific methodology. This is because their goal is to increase the understanding for age-specific physical as well as (limited) cognitive limitations of seniors. Less common is the measurement of performances by focusing on physiological parameters while utilizing an age simulation suit (e.g., Reference [21] with the age suit *GERT* (GERontologic test suit), Reference [22] AGNES, Reference [19] or MAX). According to Vieweg and Schäfer (2020), only Lauenroth et al. (2017) and Scherf (2014) additionally compare the measured physiological parameters to a reference data set of older groups of people [18]. Vieweg and Schäfer (2020) investigate whether the use of the age simulation suit GERT has an impact on 20 young test persons‘ fine and gross motor skills as well as cognitive performances. By using standard values of different age groups of older people, the authors are able to show limited evidence for the suitability of an age simulation suit for generating reference data.

The authors of this paper already dealt with the question of how to design experimental studies to generate reference data for seniors. Therefore, young and healthy participants were asked to wear the age simulation suit GERT. Within the scope of a student research with a small number of participants, first effects in the measured physiological data could be identified that refer to a group of elderly people. According to this, the participants’ performance, that was mainly measured by number of steps as well as gait speed, the study showed an increase in age of 28 to 38 years and a decrease in gait speed of 0.2 m/s while wearing GERT. The student research focused on three experimental designs: the 2-min-walk-test [23], the 6-min-walk-test (6MWT) [24] and the 10-m-walk-test [25]. The parameters were measured by making use of a stop watch as well as the fitness tracker Mi Band 2. The results show that especially the 6MWT is suitable for generating reference data using GERT.

As symptoms of chronic heart failure resemble and partially cover those of age-related impairments, the assumption is made that such a suit can be used in this context. To confirm this assumption, the method’s suitability needs to be tested under experimental conditions. Hence, the aim of this paper is to develop an experimental design that enables the use of an age simulation suit in a randomized controlled trial to generate reference data. It will focus on situations of increased physical activity with young and healthy participants to represent people with specific health impairments. Further, the study involves

(1) confirming the use of GERT as a possible way to retrieve reference data by reducing the physical performance of young and healthy participants and consequently generating pilot data; therefore, the generated data is compared with previously collected physical activity data on real patients with chronic heart failure,
(2) examining, how subjective experience and well-being influence the objective physiological parameters under two kinds of physical activity while wearing GERT and
(3) evaluating several sensors/fitness trackers regarding their usability to perform measurements to obtain a part of the reference data.

The experimental design of this paper is guided by the study conducted by Vieweg and Schäfer (2020). Therefore, in addition to answering the central research questions mentioned above a comparison of the results of both studies will be carried out. This paper is structured as follows: First, the foundations of the used age simulation suit GERT is introduced. Subsequently, the experimental design is presented, whereas the general experiment plan, inclusion and exclusion criteria for participants, precautionary measures, as well as an analysis plan is explained. The paper closes with concluding remarks and an outlook on future work.

## 2. Materials and Methods

In this section, the materials and methods required for the experimental design of the study are described. Therefore, Section 2.1 introduces the used age simulation suit, followed by Section 2.2, Section 2.3 , Section 2.4 and Section 2.5, in which the inclusion and exclusion criteria for participants, the experiment plan, questionnaires as well as the analysis plan are specified.

### 2.1. The Age Simulation Suit GERT

**Figure 1:**
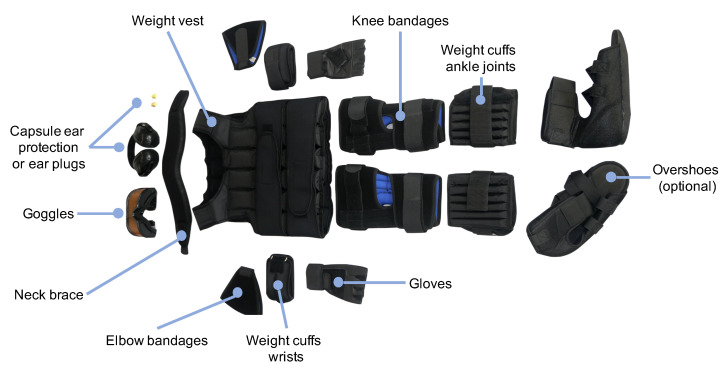
The age simulation suit GERT.

The age simulation suit GERT simulates characteristical impairments (here: limitations of the musculoskeletal system) as joint stiffness, loss of strength or impaired coordination by making use of, for example, bandages and weights [26]. In contrast to other age simulation suits presented in Section 1 GERT consists of several modules that can be flexibly combined to simulate different impairments. Furthermore, the modules allow for an unlimited size variability of participants. Figure 1 shows the basic structure of GERT consisting of a weight vest (10 kg), several bandages for the elbows and knees, weight cuffs for the ankle joints (2.3 kg each) as well as the wrists (1.5 kg each), a neck brace and gloves to reduce the mobility of the respective body parts. Furthermore, goggles and capsule ear protection or ear plugs can be used to affect the vision and simulate high frequency hearing loss. In addition to this basic equipment, the figure includes overshoes, as it affects the safety of the own walk.

### 2.2. Participants

It is planned to enroll 60 adult students aged 18 to 40 years from varying disciplines at Trier University. It is intended that the group of participants has an equal distribution of gender as well as different fitness levels. Instead of calculating the sample size, for example, with G*Power (see, e.g., Reference [27]) we have not stored a minimum sample size due to effect sizes that cannot be determined in advance. Therefore, this study serves as a systematic work to obtain a basis for further experiments in this context. Since a homogeneous group of participants is targeted, it is probable that a small number of participants already yields a significant and powerful result. As already described in Section 1, the proposed study design is inspired on the study by Vieweg and Schäfer ([18]). Here, a group of 19 individuals (mixed gender) has proven to be sufficient. As this pilot study aims to investigate some physiological and cognitive aspects, but also the usability of different fitness trackers, a significantly increased number of participants of 30 within one group (intervention or control group) and 60 in total was agreed upon.

The students have to be in self-reported good health. Excluded from participation in this study are persons according to the following description:

- persons who already have physical (i.e., cardiovascular), neurological (i.e., epilepsy) or psychological diseases,
- pregnant women,
- persons with electrical or metallic implants and
- persons with other acute diseases or impairments which might endanger their own safety, present a risk of contagion or affect their mobility.

The acquisition of test persons will take place via the university-internal e-mail distribution list as well as advertising in selected events with a large number of participants. The students sign a declaration of consent and have the chance to win one of two tablets. Furthermore, the Ethics committee of Trier University approved the study design.

### 2.3. Experiment Plan

We plan on conducting a randomized controlled trial to examine differences in physical activity performance and related psychological factors caused by wearing differently weighted GERT suits (between-subject factor: GERT with full weight vs. with low weight) in two different physical activity tasks (within-subject factor). During the experiment participants wear GERT, which is either equipped with full weight (GERT with full weight) or a significantly reduced version with only a few weights (GERT with low weight) (**between-subject**). We chose to use either the full or low GERT for each participant because otherwise they would know about the expected effect and might thus bias the study results. Both versions of the suit have the same visual appearance for the participants. The choice of full or low weight GERT is allocated randomly in a ratio of 30 to 30 participants, whereas the participants are not informed of that fact. Thus, in comparison to, for example, Reference [20], participants are not aware if they are in the intervention or control group. By giving the control group a version of GERT with low weight, measuring effects of a behavioral expectation that might unintentionally be suggested by wearing the suit is avoided. The attendant, on the other hand, is informed about the current version of GERT (*single-blinded design* [28]). Before the experiments start, the participants are informed about the experimental environment as well as the nature of the experiments‘ execution, whereas the central objective is not revealed. Instead, the participants are told, that the true nature of the study lies in the evaluation of the sensors under different levels of physical activity or exhaustion.

The study encompasses two different physical activity tasks (**within-subject**), that are conducted in a balanced order. The following paragraphs define the procedure of the experiments, that solely differ in the order of the two tasks and the equipment of the suit, that is, with full or low weight. In addition to the measurements taken with the sensors, the attendant observes the participants during the experiment. If the participants agree, the experiment is documented via video and audio recordings for further investigation.

Each participant starts with a resting period of thirty minutes in order to recover from any exertion related to, for example, sports activities or simply an arrival by bike or foot. This recovery should lead to a reduction of distortions and avoidance of increased scattering of measured values. Thereafter, the participants get into the age simulation suit and are equipped with the sensors. To protect the participants, for example, in case of falls while wearing the suit, the stairs are secured with gymnastic mats and the participants receive protective clothing for wrists, elbows, knees, shins and head. As the experiments are to be conducted in winter of 2020/2021, the situation of the Corona pandemic demands additional protective measures: in each of the tasks, the participants are asked to wear a thin protective suit before putting on GERT as well as a simple face mask. Subsequently the participants have fifteen minutes to get used to the age simulation suit without additional physical effort. During this, measurements are taken to establish a baseline for later analysis. As a next step participants perform one of the two following and randomly chosen tasks.

The first task is a *6MWT* (cf. Reference [24]). Here, the distance that participants can walk within a 6 minutes time period is measured. Therefore, a path is marked on a flat surface and the participants are instructed to walk back and forth this path until they are told that the time is over. If necessary, the participants are allowed to rest without supporting themselves, for example, on a wall [29]. The 6MWT has already been used in multiple studies focusing on people with chronic heart failure (see, e.g., Reference [29]). It has a good test-retest-reliability as well as construct validity [24]. The second task is the *stair-climb-test (SCT)*, that focuses on the participants’ “ability to ascend and descend a flight of stairs, as well as lower extremity strength, power, and balance’’ [30] (cf. References [31,32]). The test measures the number of steps that participants can climb up and down within a period of one minute. The participants in this test are allowed to pause during the task, but not to skip individual stairs or support themselves using the stair railing. The SCT shows good test-retest reliability and measurement errors in comparable studies with people having impairments (see, e.g., References [33]). The attendant adheres to an established protocol when describing tasks to prevent effects such as demand characteristics [28].

After the first randomly selected task is completed, the age simulation suit and the sensors are removed and the procedure starts over again with a recovery of thirty minutes in time. Once the same components of the age simulation suit and the identical sensors have been applied to the participant, a new baseline is established within fifteen minutes. Then the participant performs the second task that was not yet conducted.

**Figure 2:**
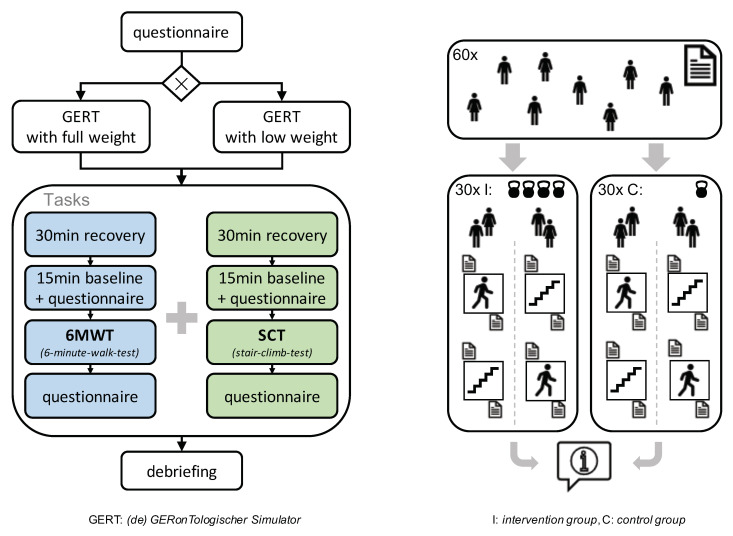
Sketches of the structure of the planned experiment.

Figure 2 shows the structure of the experiment design. The left part of the figure depicts the procedure for each participant. On the right side the randomized distribution of all participants into the two groups and the order of the two tasks is presented. To ensure equal distribution of gender as well as fitness levels to the intervention and to the experimental group, stratified randomization will be applied [34]. Accordingly, stratification characteristics will be gender and the subjective physical fitness. In order to start the experiments already in parallel to the enrollment of the study, we use the method of permitted randomized blocks [34]. Potential participants that have passed the exclusion criteria after a first screening will be processed in blocks for randomization. We will use a computer algorithm for clustering participants with respect to gender and subjective physical fitness together with a pseudo random number generator for allocating participants to intervention or experimental group within the clusters.

In order to measure relevant physiological as well as movement-specific parameters, sensors are used. Therefore, this paper utilizes the accelerometer *Movisens Move 3*, as it is optimized for the use in scientific studies [35]. An accelerometer is an “inertial sensor that can measure acceleration along its sensitive axis” [36]. The *Move 3* allows for analysis of, for example, activity classes, body position or steps [35]. In addition to this accelerometer, commercially available fitness trackers (wearables) can be utilized. Several studies, for example, Reference [37] or Reference [38], investigated the usability of fitness trackers (Samsung Gear, FitBit or Mi Band series and others) with respect to, for instance, accuracy, whereas their conclusion is mostly positive.

Before and after conducting the tasks the participants are asked to answer several questionnaires (for detailed information see Section 2.4). After completing the two tasks and the last questionnaire, each participant is informed about the details and objectives of the study.

### 2.4. Questionnaires

In addition to physical activity indicators derived from performances of the two standardized physical activity tasks and accelerometer measurements, the following self-reported outcomes will be assessed immediately before and after each task (wearing the suit), that is, at four measurement time points:

The BORG rating of perceived exertion scale (BORG RPE-scale [39]) will be used to assess *perceived exertion* while wearing the suits at rest and after completing each physical activity task. This widely used scale provides reliable estimates exertion intensity during exercise and is applicable for both healthy persons and individuals with chronic diseases [39].

Additional short-term changes in *perceived physical constitution* will be assessed with the *Wahrgenommene körperliche Verfassung* (WKV) questionnaire (cf. Reference [40]). Participants describe their actual physical state (“At the moment I’m feeling physically…”), by judging 20 adjectives covering the four dimensions “activated”(e.g., “without energy”, “drained”, all reverse scored), fitness (e.g., “powerful”, “able-bodied”), health (e.g., “healthy”, “harmed”, reverse scored), and flexibility (e.g., “agile”, “stiff”, reverse scored) on a scale from 0 (“not at all”) to 5 (“completely”). The instrument has sufficient reliability and is sensitive for changes induced by different physical activities [40].

Changes in *positive and negative affective states* will be measured via the German Positive and Negative Affect Schedule (PANAS; [41]). Participants rate how they feel “at this actual moment” by responding to 10 positive words and 10 negative words on a 5-point Likert-type scale ranging from 1 *gar nicht* [not at all] to 5 *äußerst* [extremely]. This scale has been widely used with satisfactory reliability and validity [42].

Changes in basic mood states valence, calmness, and energetic arousal will be measured via the six-item short scale developed by Wilhelm and Schöbi (cf. Reference [43]). Participants describe how they feel at this moment by responding to 6 bipolar items on a scale from 0 to 6, with both end-points labelled “very”, [for example, tired–awake (E), content–discontent (V), relaxed–tense (C)] [43]. This scale has been shown to reliably reflect changes in mood states at the between- and within-person level [43].

*Self-efficacy regarding performance of the two physical activity tasks* will be assessed applying six items (“How confident are you to be able to …”) with responses ranging from 0% to 100%. Two items pertain to the 6MWT [cover a distance as long as possible within 6 minutes (a) with short breaks and (b) without breaks], four items pertain to the 1SCT [cover as much steps as possible within 1 min, (a) upstairs using railing, (b) downstairs using railing, (c) upstairs without using railing, (c) downstairs without using railing].

Before and after completion of both tasks (wearing the suit), the domain specific *fear of physical activity* will be assessed using the Fear of Physical Activity in Situations—Heart Failure questionnaire, adapted for healthy persons. This stimulus-response inventory presents 15 situations of everyday physical activities of moderate to vigorous intensity, which are rated on a scale from 0 (“low”) to 5 (“high”) in terms of both cognitive worry and feelings of tension. Both the original heart failure version and the adaptation for healthy persons have shown good reliability (Cronbach’s α = 0.98 and 0.97 respectively) and findings from both populations indicate the instrument’s validity [44,45,46].

Additional assessments include *demographic characteristics* (age, gender, education, working for an income), anthropometric variables (weight, height, blood pressure), and variables that might also be associated with physical activity task performance and which will be used as control variables, such as smoking (never, past smoker, current) and participants’ usual *level of physical activity*. The latter will be assessed using the *Bewegungs- und Sportaktivität Fragebogen* (BSA-F; [47]). Three scores can be calculated that reflect duration of (1) activities that are performed while at work or during leisure time including stair climbing, (2) sports activities, and (3) total physical activity, each in minutes/week during the past 4 weeks. Indicators of satisfactory validity have been reported [47]. The German version of the Mainz Coping Inventory Physical Threat subtest will be used to assess the coping dispositions vigilance and cognitive avoidance in situations of physical threat (MCI [48]). The MCI has been shown to provide a reliable and valid measure of these two central coping dimensions [49].

The German version of the Outcome Expectancies for Exercise Scale (OEE-2) is a 13 item questionnaire to assess positive and negative outcome expectancies related to engaging in physical activities in older adults (cf. Reference [50]). The instruments has shown adequate reliability (Cronbach’s α = 0.89) and validity in geriatric patients [50].

### 2.5. Analysis Plan

The research focuses on three hypotheses that covers the materials (GERT, questionnaires) and the relationships to the physical activity parameters under investigation:

**Hypothesis** **1** **(H1).** *Wearing the age simulation suit with full weight negatively influences the participant’s physical activity.*

**Hypothesis** **2** **(H2).** *A high self-reported fitness level positively moderates the negative relationship between wearing an age simulation suit and physical activity.*

**Hypothesis** **3** **(H3).** *A high level of fear of activity negatively influences the participant’s physical activity.*

Analyses will be conducted using R [51]. To test whether randomization was successful demographic and baseline characteristics will be compared using t-tests and chi square tests as appropriate. Effects of GERT with full and low weight on performance of each physical activity task and associated psychological characteristics will be analyzed using a series of ANOVAS. Furthermore, it is examined whether the task 6MWT generates similar results as in other studies (e.g., Reference [18]). Differences in measurements and perceptions before and after performing the two tasks (6MWT and SCT) will be identified via repeated measures ANOVA. The existence of gender-specific differences as well as the impact of (subjectively perceived) fitness levels on the participants’ performance will be computed via mixed-design analyses of variance (mixed ANOVA) with gender and fitness levels as between-subjects factors and the performed task as within-subjects factor.

The participants sign a declaration confirming their consent to the storage and use of personal data for scientific evaluation and in compliance with the data protection guidelines. The data of the participants are pseudonymized before the analysis. The participants can additionally agree, if video recording of the tasks can be carried out. The video material is to be evaluated exclusively using artificial intelligence methods. In the video recordings, the faces are pixelated using automated processes and thus pseudonymized. Manual inspection will only take place in individual cases for troubleshooting in the event of conspicuous measured values.

## 3. Conclusions

In this paper, we introduced an experimental design for a randomized controlled trial using an age simulation suit. The aim of this trial is to generate reference data with young and healthy participants representing people with health impairments, that is, people with chronic heart failure. The age simulation suit GERT will be applied, as prior work by other researchers has shown, that it is a reasonable tool for our trial. Data gathering to measure physical activity will be performed by an advanced accelerometer (Move 3) as well as several common fitness trackers. Based on our experimental design, we will contribute to the field of designing and testing assistance approaches in health care and in supporting the delay of care dependency in the long term.

Nevertheless, the experimental design has some limitations. First, due to the finite functionalities of the used materials (i.e., fitness trackers), we use a limited number of physical parameters to determine the physical exhaustion of the participants. Hence, heart rate and acceleration are the main indicators for the participants’ condition in our study. Furthermore, we use subjectively gained information about, for example, the fitness levels as well as the subjective well-being using questionnaires. However, further objective parameters, for example, the concentration of blood oxygen might be important indicators (see, e.g., Reference [52]). Second, in order to prevent the participants from potential injuries while wearing GERT, they are equipped with multiple protectors (see Section 2.3). However, these protectors might additionally restrict the participants’ movement and therefore manipulate the effect of GERT. Furthermore, the Corona pandemic makes the use of face masks as well as one-way protective suits necessary, which might impact the results as well. To encounter these impacts, we provide uniform equipment for each participant. Lastly, the study focuses on two typical situations of persons with chronic heart failure. To generate reliable reference data, the study should be extended to further everyday tasks (see, e.g., Reference [18]).

## 4. Outlook

As a next step, we will conduct the experiments and evaluate the trial as described above. Additionally, a comparison of our resulting data with real-world data of people with health impairments will be performed. Doing so, we will decide on the question, whether or not this suit and the experimental set-up is adequate for generating reference data of elderly people’s activities with students as proxy. In future work, we will extend our investigations to other activities of everyday life, in order to explore innovative assistance approaches in home health care (see, e.g., References [53,54,55,56]).

## Data Availability

Data sharing not applicable.
